# Distribution of plasma copeptin levels and influence of obesity in children and adolescents

**DOI:** 10.1007/s00431-020-03777-3

**Published:** 2020-08-18

**Authors:** Gerdi Tuli, Jessica Munarin, Daniele Tessaris, Silvia Einaudi, Patrizia Matarazzo, Luisa de Sanctis

**Affiliations:** 1 Department of Pediatric Endocrinology, Regina Margherita Children’s Hospital, Turin, Italy; 2 Department of Public Health and Pediatric Sciences, University of Turin, Turin, Italy; 3 Turin, Italy

**Keywords:** Copeptin, Distribution, AVP related disorders, Pediatric age, Obesity

## Abstract

In recent years, a more stable AVP surrogate, called copeptin, has been used as an adjuvant diagnostic tool for dysnatremia in adults and appears to be promising even in the pediatric age. The aim of this study is to present the distribution of plasma copeptin in a large pediatric cohort and to observe the influence of fluid consumption and obesity on its values. A cohort of 128 children and adolescents was divided into two groups on the basis of nocturnal deprivation (group A) or free access to oral fluids in the 6–8 h before blood collection (group B). At all distribution percentiles, copeptin levels were higher (*p* < 0.0001) in group A, as were plasma sodium levels and osmolality (*p* = 0.02 and *p* = 0.008, respectively). The influence of BMI on copeptin levels was investigated by dividing the cohort into nonobese (group C) and obese children and adolescents (group D). Copeptin levels were higher in group D (*p =* 0.04).

*Conclusion*: The measurement of copeptin could represent a useful tool for the diagnostic pathway of dysnatremic conditions, but its interpretation should take into consideration the state of hydration. Furthermore, it could also be a promising marker for obesity and metabolic syndrome, although this hypothesis needs further studies to be confirmed.

**Table:** 

**What is Known:** • *Copeptin use as a diagnostic tool in AVP-related disorders, such as diabetes insipidus or syndrome of inappropriate secretion of antidiuretic hormone, is well established in adults* • *In pediatric age, few studies are available, but the preliminary data, including our previous study, seems to be promising.*	
**What is New:** • *In this study, we represent the distribution of copeptin levels in a pediatric cohort and show the significant influence of fluid ingestion on its plasma levels.* • *Also BMI seems to be a significant variable on copeptin levels and may be used as an obesity marker in pediatric age*

**What is Known:**

• *Copeptin use as a diagnostic tool in AVP-related disorders, such as diabetes insipidus or syndrome of inappropriate secretion of antidiuretic hormone, is well established in adults*

• *In pediatric age, few studies are available, but the preliminary data, including our previous study, seems to be promising.*

**What is New:**

• *In this study, we represent the distribution of copeptin levels in a pediatric cohort and show the significant influence of fluid ingestion on its plasma levels.*

• *Also BMI seems to be a significant variable on copeptin levels and may be used as an obesity marker in pediatric age*

## Introduction

Arginin-vasopressin (AVP) or antidiuretic hormone (ADH) is one of the main hormones involved in the regulation of water and sodium homeostasis [1–10]; AVP-related disorders are therefore a heterogeneous group of conditions often characterized by severe symptoms, mainly related to dehydration and dysnatremia, even potentially life-threatening if not promptly recognized and adequately treated.

Hyponatremia associated with an inappropriate AVP secretion characterizes the syndrome of inappropriate antidiuretic hormone secretion (SIADH); its diagnosis is often challenging, since its biochemical features overlap with those of the cerebral/renal salt wasting syndrome (C/RSW), a rare and controversial but severe hyponatremic condition found mainly in patients with cerebral tumors [11–16].

Conversely, hypernatremia is the common finding of an insufficient AVP secretion, as observed in central diabetes insipidus (CDI), or an insufficient AVP action despite increased release, in case of AVP receptor resistance (nephrogenic diabetes insipidus, NDI) [17–20].

Plasma concentration of AVP has been classically used in the diagnostic process of all these disorders, but its structural instability, its very short plasma half-life and, last but not least, the long-lasting laboratory processing have limited its use in clinical practice [6, 17–31].

Recent studies have evaluated the usefulness of plasma copeptin in the differential diagnosis of hyponatremic and hypernatremic AVP–related disorders, as it represents a stable and rapidly available surrogate of plasma AVP [32–34]. It has proven to be very useful in the distinction of hypernatremic conditions (such as CDI, NDI, and PP) and less useful in hyponatremia due to the important overlap between SIADH, hyper- or hypovolemic hyponatremia [35–47], a finding already reported also in a small number of pediatric patients [42]. In adults, it seems to be a promising diagnostic tool even after stimulation tests with hypertonic saline or arginine, not yet explored in children [48, 49].

In children, plasma copeptin has been considered as a possible prognostic index of some conditions, such as septic shock, pneumonia, stroke, heart and kidney failure, and traumatic brain injury [50–60]. Higher copeptin levels have also been described in insulin resistant obese children [61]. However, to date few data exist on the distribution of plasma copeptin in the pediatric age and indicate a range between 2.4 and 8.6 pmol/L [8, 42]. Our experience on 53 children not affected by AVP-related disorders showed a significant difference in copeptin levels between subjects with free access to fluids and subjects with restriction to fluids for at least 6–8 h [42].

The purpose of this study is to present the distribution of plasma copeptin in a larger pediatric cohort, also considering fluid intake and body mass index (BMI).

## Material and methods

### Study population

Plasma copeptin levels were measured in 128 children and adolescents without AVP-related disease, who referred to the Department of Pediatric Endocrinology of the Regina Margherita Children’s Hospital in Turin in the period July 2016–May 2018.

The reason for hospitalization was a suspected puberty or short stature without hematologic or hormonal abnormalities at baseline evaluation and dynamic hormone assessment; no patient had other comorbidities. Exclusion criteria were the presence of hypo- or hypernatremia, abnormal urinary or plasma osmolality, type I diabetes mellitus, hypo- or hypercalcemia, hypokalemia, hypo- or hyperthyroidism, hypo- or hypercorticism, kidney and heart diseases, recent episodes of nausea or vomiting, infectious diseases, traumatic brain injury, enuresis, and any treatment that interferes with the AVP release system.

Since all subjects had been referred to our department for a fasting blood sample, plasma samples for copeptin investigations were collected early in the morning; based on the retrospective anamnesis of home fluids intake in the 6–8 h before sample collection, the cohort was then divided into two groups, group A without overnight fluid intake and group B subjects with free access to liquids (which was up to a maximum of 300 ml of water).

The main anthropometric parameters of height, weight, and body mass index (BMI) were detected in the whole study population and allowed the distinction between nonobese (BMI < 95th percentile, group C) and obese children and adolescents (BMI > 95th percentile, group D), accordingly to Cole’s BMI percentiles [62].

Before the start of the study, ethical approval was obtained from the Ethics Committee of the City of Health and Science University Hospital of Turin and written informed consent from the families.

### Laboratory samples for plasma copeptin

Plasma samples for copeptin evaluation were collected into tubes containing ethylenediaminetetraacetic acid (EDTA). The peptide measurement was evaluated concomitantly to serum osmolality by an immunoluminometric assay (BRAHMS CT-proAVP LIA; B.R.A.H.M.S. GmbH, Hennigsdorf Germany) with detection limit < 0.4 pmol/L and functional assay sensitivity < 1 pmol/L. The percentage of intra- and interassay coefficient of variation (CV%) was < 8% and < 10%, respectively [8].

### Statistical analysis and graphs

Statistical analysis and graphs were performed by Graphpad 7 software (GraphPad Software, La Jolla, CA, USA) using the Student *t* test for the means comparison. To assess that potential confounders did not affected the results, a multivariate analysis was also performed for BMI and age.

## Results

### Distribution of plasma copeptin levels in pediatric age

In the 128 children and adolescents (median age 8.6 years, range 1.13–17.4 years; 42 boys, 86 girls) with normal plasma sodium and osmolality, the median plasma copeptin level was 7.6 pmol/L (range 2–14.9 pmol/L).

The demographic and biochemical features of the studied population, divided into group A (spontaneous fluids deprivation for at least 6–8 h) and group B (free access to fluids) are represented in Table 1. No significant difference was observed for sex and pubertal stage between the two groups.

**Table 1:** Demographic and biochemical features of the studied population; group A (fluid fasting for at least 6–8 h) and group B (free access to fluids). Median values are reported in parentheses

	Group A	Group B	*P* value
Whole population (*n*)	40	88	
Males (*n*)	15	27	0.54
Females (*n*)	25	61	
Pubertal subjects (*n*.)	18	35	0.69
Pre-pubertal subjects (*n*)	22	53	
Age (years)	8.56 (1.13–17.4)	8.66 (2.23–16.9)	0.61
Plasma sodium (mmol/L)	141 (138–145)	140 (134–145)	0.02
Plasma osmolality (mOsm/kg)	285 (276–291)	283.5 (276–315)	0.008
Plasma copeptin (pmol/L)	10.6 (3.3–14.9)	4.9 (2–10.8)	< 0.0001

**Fig. 1:**
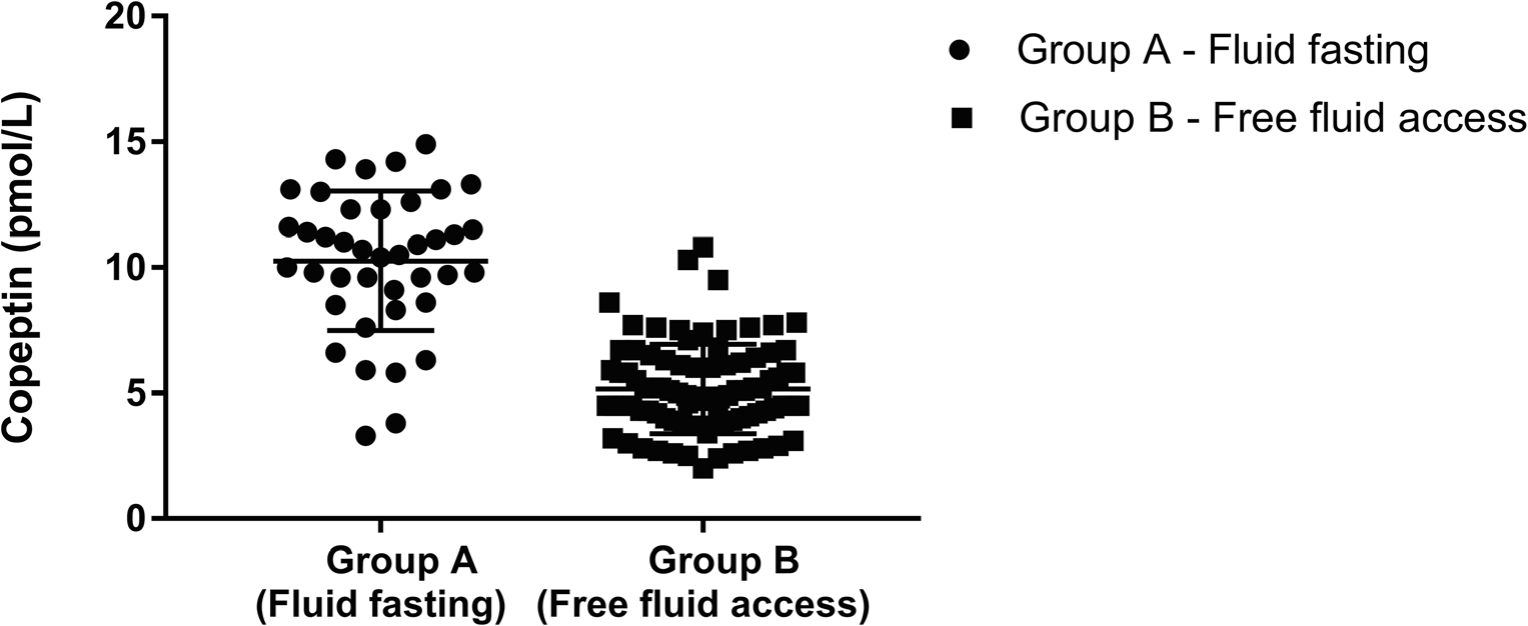
Plasma copeptin levels after fluid fasting (group A) and free access to fluids (group B)

The median plasma copeptin level in group A was 10.6 pmol/l (range 3.3–14.9), while in group B 4.9 pmol/L (range 2–10.8) (*p <* 0.0001), as shown in Fig. 1.

The median plasma sodium level was 141 mmol/L (range 138–145) in group A, while 140 mmol/L (range 134–145) in group B (*p* = 0.02). A significant difference between the 2 groups was also observed for plasma osmolality (median 285 mOsm/kg (range 276–291) in group A vs 283.5 mOsm/kg (range 276–315) in group B, *p =* 0.008).

The distribution in percentiles of the plasma copeptin for each group is shown in Table 2.

**Table 2:** Distribution of plasma copeptin in percentiles

Percentiles	Whole population (pmol/L) (*n* = 128)	Group A (pmol/L) (*n* = 40)	Group B (pmol/L) (*n* = 88)
3th	2.59	3.42	2.47
5th	2.7	3.9	2.6
10th	3.19	5.94	2.8
25th	4.43	8.73	4
50th	5.85	10.6	4.9
75th	9.4	12.3	6.18
90th	11.51	13.84	7.6
95th	13.1	14.3	8.24
97th	13.94	14.76	9.76

No difference was present between males (*n* = 42) and females (*n* = 86), for which median copeptin level was 7.1 pmol/L (range 3.3–11.2) vs 6.7 pmol/L (range 2–14.9) respectively (*p = 0.61*).

### Copeptin levels in obese children and adolescents

Biochemical data of nonobese (group C, 102 children, 28 males and 74 females, age 8.35 years, range 1.13–17.4 years) and obese subjects (group D, 26 children, 14 males and 12 females, age 9.92 years, range 3.35–16.7 years) are represented in Table 3.

**Table 3:** Plasma sodium, osmolality and copeptin levels in not obese and obese children. Median values are reported in parentheses

	Group C (not obese)	Group D (obese)	*P* value
Whole population (*n*)	102	26	–
Males (*n*)	28	14	0.26
Females (*n*)	74	12	
Pubertal subjects (*n*)	38	15	0.54
Prepubertal subjects (*n*)	64	11	
Spontaneous fluids deprivation (*n*)	29	11	0.4
Fluids restriction (*n*)	73	15	
Age (years)	8.35 (1.13–17.4)	9.92 (3.35–16.7)	0.02
Plasma sodium (mmol/L)	141 (134–145)	140.5 (138–145)	0.44
Plasma osmolality (mOsm/kg)	284 (276–315)	284 (276–297)	0.47
Plasma copeptin (pmol/L)	5.5 (2–14.9)	7.15 (3.1–14.2)	0.04

**Fig. 2:**
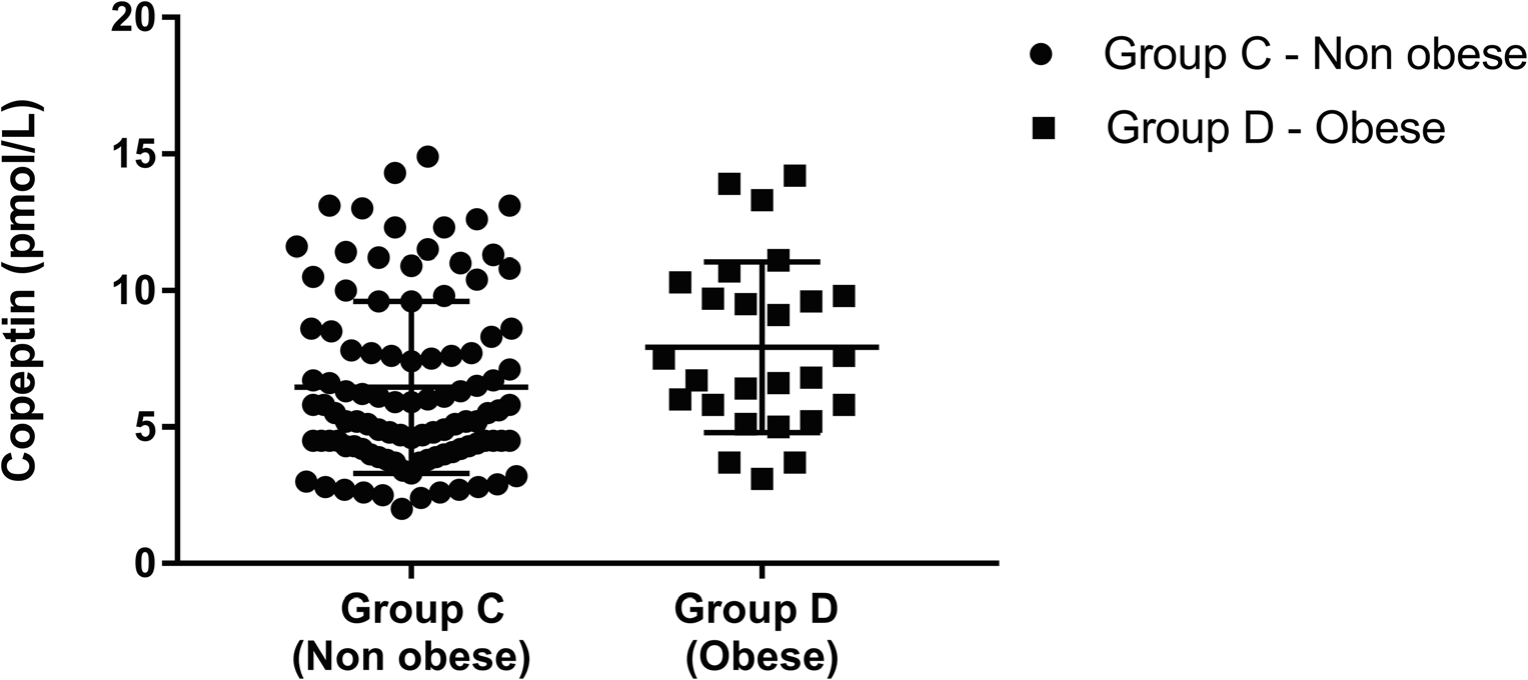
Plasma copeptin levels in non-obese children (group C) and obese children (group D)

Plasma copeptin level was 5.5 pmol/l (range 2–14.9) in group C, 7.15 pmol/L (range 3.1–14.2) in group D (*p =* 0.04), as shown in Fig. 2.

The multivariate analysis considering BMI showed no significant differences between the two groups for sex, pubertal stage, fluid state, plasma sodium, and osmolality, but higher age for the obese group (*p* = 0.02). The multivariate analysis considering the age indicates a significant higher proportion of prepubertal females in the nonobese group (*p* < 0.001), but Pearson’s correlation coefficient between copeptin levels and age was 0.04 (*p* = 0.63), and no difference was found in copeptin levels between males and females in the whole studied population (*p* = 0.43), as well as in the obese cohort (*p* = 0.78).

## Discussion

AVP-related disorders include several clinical conditions that can be life-threatening when hyponatremia or hypernatremia become severe.

Therefore, measurement of plasma AVP may be useful in the diagnostic pathway of all these disorders, but its in vitro instability and short half-life have made this investigation almost useless. In recent years, plasma level of an AVP surrogate, called copeptin, has been used thanks to its higher stability and fewer preanalytical procedures [6, 29–32].

Copeptin plasma levels in healthy adults range from 1 to 13 pmol/L and are very sensitive to osmotic and hemodynamic stimuli similarly to AVP [48, 63, 67]. Males show slightly higher copeptin values with minimal difference in median values; age or circadian rhythm does not seem to interfere nor does food intake or menstrual cycle.

In adults, several studies have evaluated the usefulness of copeptin in the diagnostic pathways of AVP-related diseases, as well as its potential role as prognostic marker in some clinical conditions such as acute myocardial lesions, renal and heart failure, stroke, and sepsis [7, 9, 30–41, 43–47].

Despite many authors have established its usefulness in adult clinical practice, few data have been produced on the value of copeptin as a diagnostic tool for AVP-related diseases in children. Overall, few data exist on the distribution of plasma levels in children and adolescents [42, 50–61]. In a previous paper, we have shown the distribution of copeptin plasma levels in 53 children and adolescents without AVP-related diseases, indicating ranges between 2.4 and 8.6 pmol/L for the pediatric age [42]; in the present study, we have expanded our cohort of controls without any AVP-related diseases to establish a more defined distribution in the evolutive age.

Plasma copeptin levels were measured in a cohort of 128 children and adolescents who were divided into two groups on the basis of anamnestic fluid ingested in the 6–8 h prior to collection; the first group included children without fluid intake for at least 6–8 h before the sample collection, while the second one children with free access to fluids. Copeptin, sodium, and osmolality levels differed significantly in the two groups, although sodium and osmolality were in the normal reference range in both groups. This fact reinforces the knowledge that even small fluctuations of sodium and osmolality within the normal reference range can lead to significant changes in the release of AVP, a peptide that is really sensitive to the hydration status [9, 32–34]. Subjects with free access to fluids showed lower values in all percentiles of the copeptin levels than subjects with fluid deprivation, highlighting that oral fluid intake, even in small quantities, leads to a significant decrease in copeptin values and should be taken into account in the interpretation of its plasma levels. Previous studies have indicated mean normal copeptin levels similar to those reported here, without defining a percentile distribution [50, 52, 54, 58].

We are aware that a major limitation of this study is represented by the size of the cohort which allowed us to indicate only the distribution, as it should be much larger to obtain true reference percentiles. Another limitation can be represented by the evaluation of the hydration state at the time of the sample collection, since it is known that the copeptin is very sensitive to the variation of even small quantities of liquids, both administered intravenously and ingested orally. So we decided to divide the series of cases into subjects without any fluid intake during the night preceding the collection and subjects who had taken it, even if in small quantities.

The relationship between copeptin levels and obesity [64–69], as well as its association with metabolic and cardiovascular risk factors, has already been explored in adulthood and in animal models. A positive correlation between plasma copeptin levels and metabolic syndrome parameters had already been described also in children, and higher copeptin levels have been described in obese children [60, 61]. Our data, within a cohort of subjects with normal serum sodium and osmolality, indicate significantly higher copeptin levels in obese children than in the nonobese counterpart. The significant differences in the age of the two groups could be explained by the fact that the onset of obesity occurs mainly in adolescence, even if copeptin levels did not show any correlation to age nor difference for sex. Therefore, plasma copeptin levels might be an important indicator of BMI-related glucose metabolism dysfunction, although further studies with larger cohorts and analysis of metabolic parameters are needed to confirm this hypothesis.

## Conclusion

The present study further strengthens the fact that plasma copeptin level could be regarded as a useful indicator of AVP system function and water and sodium homeostasis; it should therefore be included in the diagnostic pathway of all hypernatremic and hyponatremic conditions for a more correct diagnosis and consequently an appropriate treatment choice.

It is undoubtedly a marker very sensitive to plasma sodium and osmolality variations, as well as to fluids administered orally or intravenously, which therefore must be considered in the interpretation of the results.

In the present study, we presented the distribution of plasma copeptin in percentiles in a population of children without disturbances of the AVP release system, in order to have normal copeptin values as potential reference in the suspicion of AVP release disorders.

Considering the anthropometric variables in our control population, we can postulate that copeptin might also be a promising marker for other conditions such obesity and the metabolic syndrome in children and adolescents, although this hypothesis needs further studies to be confirmed.

## Abbreviations

ADH: Antidiuretic hormone
AVP: Arginin-vasopressin
BMI: Body mass index
CDI: Central diabetes insipidus
C/RSW: Cerebral renal salt wasting syndrome
NDI: Nephrogenic diabetes insipidus
SIADH: Syndrome of inappropriate antidiuretic hormone secretion
WDT: Water deprivation test
